# Clinical and Laboratory Analysis of Patients with Leishmaniasis: A Retrospective Study from a Tertiary Care Center in New Delhi

**Published:** 2017

**Authors:** Nitin GUPTA, Kamla KANT, Bijay Ranjan MIRDHA

**Affiliations:** 1. Dept. of Medicine and Microbiology, All India Institute of Medical Sciences, New Delhi, India; 2. Dept. of Microbiology, All India Institute of Medical Sciences, New Delhi, India

**Keywords:** Visceral leishmaniasis, Cutaneous leishmaniasis, PKDL

## Abstract

**Background:**

Leishmaniasis manifests as visceral (VL), cutaneous (CL) or a dermal sequel of VL, known as Post kala-azar dermal leishmaniasis (PKDL). The aim of the study was to analyze the clinical and laboratory features of cases diagnosed with leishmaniasis.

**Methods:**

This hospital-based retrospective study included all cases of VL, PKDL, and CL diagnosed between Jan 2011 to Jan 2016 at All India Institute of Medical Sciences, New Delhi. Clinical and laboratory profile of the diagnosed cases were analyzed in detail. All diagnosed cases were mapped according to the state and the district from which the cases originated.

**Results:**

A total of 91 VL cases and 4 PKDL cases were reviewed. Only one case of CL (1 female) and mucocutaneous leishmaniasis (1 female) were observed during the study period. Majority of the cases of VL (75/91) originated from Bihar. The most common presenting symptoms in all our patients were fever (97.8%), weight loss (40.6%) and abdominal discomfort (17.6%) while the most common presenting signs were hepatosplenomegaly (45.8%), isolated splenomegaly (23.1%) and skin pigmentation (11%). The most common laboratory abnormality was anaemia followed by thrombocytopenia and leucopenia.

**Conclusion:**

VL is globally recognized as a neglected tropical disease. Even after continued effort to bring down its transmission in India, it continues to affect the endemic states with reports from new pockets.

## Introduction

Leishmaniasis, a group of chronic debilitating disease, largely affects the individuals living in developing countries where the disease is endemic (1). It is caused by *Leishmania* spp. transmitted by the vector Phlebotomine sand flies. The disease manifests as visceral (VL), cutaneous (CL) or mucocutaneous leishmaniasis. The two common species that are responsible for visceral leishmaniasis throughout the world are *L. donovani* and *L. infantum*. *L. donovani* causes disease in Southeast Asia and East Africa while *L. infantum* causes disease in the Mediterranean, the Middle East and Brazil (2).

In India, Visceral leishmaniasis, also known as kala-azar is mainly reported from the states of Bihar, Jharkhand, West Bengal and eastern part of Uttar Pradesh (2). Cutaneous leishmaniasis primarily occurs in the American region, Mediterranean basin, Western Asian and Central Asian region (2), however episodic cases have been reported from India, mainly from Himachal Pradesh and Rajasthan (3, 4). Rare cases of mucocutaneous leishmaniasis have been reported from India (5–7). A dermal sequel of VL, known as Post kala-azar dermal leishmaniasis (PKDL) is reported from the Indian subcontinent and Sudan (8). The mainstay in the diagnosis of leishmaniasis is by demonstration of amastigote forms (LD bodies) or by using various serological tests including point of care tests like the strip based rK39 test (9). The present study is a retrospective analysis of the clinical and epidemiological features of leishmaniasis cases diagnosed in our tertiary care center.

## Methods

This hospital-based retrospective study included all cases of visceral leishmaniasis, PKDL and cutaneous leishmaniasis diagnosed over half a decade between Jan 2011 to Jan 2016, at All India Institute of Medical Sciences, New Delhi. All patients suspected of visceral leishmaniasis who were positive for rk-39 antibody were included in the study. All parasitologically confirmed cases of PKDL and cutaneous or mucocutaneous leishmaniasis by biopsy from the lesion were included in the study. Clinical and laboratory profile of the diagnosed cases were analyzed in detail. All diagnosed cases were mapped according to the state and the district from which the cases originated. Patients residing in the non-endemic areas at the time of presentation were only considered belonging to that area if they did not have any visit to the known endemic areas prior to the onset of clinical manifestations. All care was taken to maintain the confidentiality of the patients. This was a retrospective analysis of records and therefore, consent could not be taken.

Clinical and laboratory profiles of all the cases were expressed in percentage. The data were analyzed using SPSS software (version 21.0, SPSS, Inc., Chicago, IL, USA).

The confidentiality of the subjects was maintained while analyzing the data. The study was approved by Ethics Committee of the university.

## Results

Overall, 91 visceral leishmaniasis cases (61 male, 30 female) and 4 PKDL cases (3 female, 1 male) were reviewed. Only one case of cutaneous leishmaniasis (1 female) and mucocutaneous leishmaniasis (1 female) were observed during the study period.

**Fig. 1:**
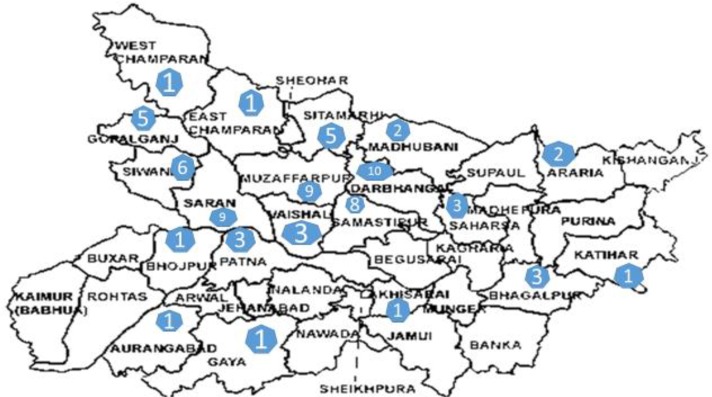
Visceral leishmaniasis case distribution in Bihar

**Fig. 2:**
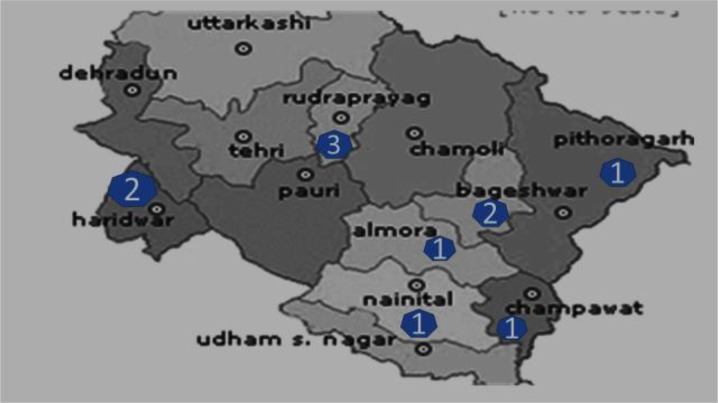
Visceral leishmaniasis case distribution in Uttarakhand

Majority of the cases of visceral leishmaniasis (75/91) originated from Bihar, and the rest (16/91) were from the adjoining states of Uttarakhand (11/91), Uttar Pradesh (3/91), and West Bengal (2/91). The district wise case distribution in Bihar and Uttarakhand is summarised in Figs. 1 and 2. From the state of Uttar Pradesh, one case was from Aligarh and two were from Agra. In West Bengal, two cases were reported from Burdwan district.

Intermittent fever was observed in almost all the cases of visceral leishmaniasis except in two patients where no history of documented fever was available. The mean duration of period in which patient had intermittent fever was 99 d. History of kala-azar was present in 4 cases. Four patients received empirical Anti-tubercular therapy (ATT) before the diagnosis of VL was made.

The clinical and laboratory profile of visceral leishmaniasis cases are summarised in Table 1. All the patients were treated with intravenous Amphotericin B (liposomal or deoxycholate).

**Table 1:** Clinical and laboratory profile of Visceral leishmaniasis cases

***Clinical and Laboratory profile***	***Frequency (n=91)***
Significant weight loss	37 (40.6)
Cough	5 (5.5)
Diarrhoea	5 (5.5)
Melena	2(2.2)
Bleeding manifestations	2 (2.2)
Abdominal distension	14 (15.4)
Abdominal discomfort	16 (17.6)
Jaundice	3 (3.3)
Skin pigmentation	10 (11)
Peripheral oedema	6 (6.6)
Lymphadenopathy	1 (1.1)
Hepatomegaly	44 (45.8)
Splenomegaly	65 (71.4)
Hepatosplenomegaly	44 (45.8)
Anaemia	59 (64.8)
Leucopenia	34 (37.4)
Thrombocytopenia	48 (52.7)
Pancytopenia	34 (37.3)
Albumin: Globulin reversal	22 (24.2)
Transaminitis	23 (25.3)

The only case that presented with cutaneous leishmaniasis from Himachal Pradesh (Kin-naur district) presented with a plaque on cheek and neck with no previous history of kala-azar. She had pancytopenia with deranged liver function tests (LFT). She was diagnosed by skin biopsy. One case of mucocutaneous leishmaniasis from Uttarakhand (Pithoragarh district) was included in our series. This patient presented with ulcer on both lips and buccal mucosa. She had pancytopenia and was positive by both rk39 positivity and direct demonstration from ulcer biopsy. All four patients who presented with PKDL were from Bihar, two each from the districts of Muzaffarpur and Sitamarhi. Three patients presented with hypopigmented macules while one patient presented with erythematous papules.

## Discussion

Most of the visceral leishmaniasis cases were reported from the states of Bihar and Uttarakhand. Visceral leishmaniasis is not known to be endemic in New Delhi but it is home to a large number of migrant workers mainly from neighboring areas of Bihar, Uttar Pradesh, and West Bengal. Several of the cases were residing in New Delhi at the time of presentation but had frequent visits to their native places that are endemic to leishmaniasis. These cases were, therefore, not identified to be originating from New Delhi. Moreover, since ours is a tertiary care referral center, we receive patients of all specialties from all across North India on a regular basis. Although Kala-azar is classically reported from the plains of eastern India, we saw a surprisingly high number of cases from the sub-Himalayan region of Uttarakhand. There has been a recent upsurge in the number of cases reported from the Uttarakhand region especially from the Kumaon and Garhwal district (10, 11). The single case of cutaneous leishmaniasis was reported from Kinnaur, Himachal Pradesh, which is one of the biggest foci of cutaneous leishmaniasis in India (3). PKDL in India is reported in around 15% of cases of visceral leishmaniasis, mainly from the endemic areas of Bihar (12). As expected, all four cases who presented with PKDL were from Bihar.

The most common presenting symptoms in all our patients were fever (97.8%), weight loss (40.6%) and abdominal discomfort (17.6%) while the most common presenting signs were hepatosplenomegaly (45.8%), isolated splenomegaly (23.1%) and skin pigmentation (11%).

The most common laboratory abnormality was anaemia followed by thrombocytopenia and leucopenia. Pancytopenia was seen in around 37% of cases. The infiltration of parasite by bone marrow causes suppression of cell lines leading to pancytopenia (13). Transaminitis and Albumin globulin ratio reversal was seen in 25% and 24% cases respectively. Several studies from India in the past have shown the following range of frequencies of clinical features and laboratory abnormalities in diagnosed cases of visceral leishmaniasis such as: fever (70%–100%), splenomegaly (85%–100%), hepatomegaly (40%–100%), anaemia (94.4%–100%), leukopenia (38.8%–100%), thrombocytopenia (33.3%–100%) and pancytopenia (58.3%–100%) (10, 11, 14–16). In comparison, the following observations were noted from studies on patients with visceral leishmaniasis in Iran, where *L. infantum* is common: fever (96.9%), splenomegaly (91.5%), hepatomegaly (53.6%), lymphadenopathy (21.1%) and Anaemia (99.5%) (17, 18).

The rk 39 antibody detection test uses the namesake antigen, encoded by the kinesin-related gene is used to detect antibody in patient’s serum (19). It has been found to be highly sensitive and specific in Indian patients with VL (20). All patients of visceral leishmaniasis were, therefore, primarily diagnosed based on serum rk39 only. Since the sensitivity of antibody-based tests is poor in case of cutaneous or mucocutaneous leishmaniasis due to poor humoral response, they were diagnosed by direct demonstration of LD bodies in skin biopsy (21). Liposomal amphotericin B is the recommended anti-leishmanial drug for use in India with high cure rates. The usual doses are 3–5 mg/kg once daily for a total of 3–5 d or 10 mg/kg as a single dose by infusion (22). Deoxycholate amphotericin B for 15–20 doses is used as an alternative in low resource settings due to high cost of liposomal amphotericin B (22).

## Conclusion

Visceral leishmaniasis is globally recognized as a neglected tropical disease. Even after continued effort to bring down its transmission in India, it continues to affect the states of Bihar, Jharkhand, West Bengal an Uttar Pradesh. In addition, the disease continues to be reported from new pockets. In the presence of suggestive clinical and laboratory features, appropriate diagnostic tests should be carried out. In addition, cutaneous leishmaniasis should be kept in differential in patients from endemic areas with clinically suggestive lesions.
